# Suppression Rather Than Visual Acuity Loss Limits Stereoacuity in Amblyopia

**DOI:** 10.1167/iovs.61.6.50

**Published:** 2020-06-24

**Authors:** Ann L. Webber, Katrina L. Schmid, Alex S. Baldwin, Robert F. Hess

**Affiliations:** 1 School of Optometry & Vision Sciences and Institute of Health & Biomedical Innovation, Queensland University of Technology (QUT), Kelvin Grove, Queensland, Australia; 2 Department of Ophthalmology & Visual Sciences, McGill University, Montreal, Quebec, Canada

**Keywords:** stereopsis, suppression, stereoacuity, amblyopia, visual acuity

## Abstract

**Purpose:**

To investigate the influence of interocular suppression and visual acuity loss on stereoacuity in observers with and without abnormal vision development from strabismus or amblyopia. To determine whether stereoacuity improves in amblyopic observers when suppression is neutralized.

**Methods:**

Experiment 1: Visual acuity (VA), depth of suppression (contrast ratio [CR]), and stereoacuity (digital random-dot) were tested in adult amblyopic observers (n = 21; age 27 ± 11 years). Experiment 2: VA, stereoacuity, and CR were measured at baseline and through a series of monocular contrast attenuation and Bangerter filter conditions that degrade visual input in participants with normal binocular vision (n = 19; age 31 ± 13 years). Multiple regression models were used to determine relative contribution of VA and CR to stereoacuity in both groups. Experiment 3: stereoacuity was retested in a subsample of amblyopic observers (n = 7) after contrast reduction of the stimulus presented to dominant eye to neutralize suppression.

**Results:**

In amblyopic observers, stereoacuity significantly correlated with CR (*P* < 0.001), but not with interocular VA difference (*P* = 0.863). In participants with normal vision development, stereoacuity, VA, and CR declined with introduction of monocular Bangerter filter (*P* < 0.001), and stereoacuity reduced with monocular attenuation of stimulus contrast (*P* < 0.001). Reduction in stereoacuity correlated with both VA decrement and degraded CR. Stereoacuity significantly improved in amblyopic observers when the contrast to the dominant eye was adjusted based on the contrast ratio.

**Conclusions:**

Suppression rather than visual acuity loss limits stereoacuity in observers with abnormal vision development. Stereopsis can be improved when interocular sensory dominance is neutralized.

Amblyopia is a developmental disorder of the visual system in the brain. In most cases, patients suffering from amblyopia will have reduced visual acuity in one eye. There is a degree to which the brain suppresses (or ignores) the visual signal from that eye. This typically results in reduced or absent stereopsis (binocular depth perception). Stereopsis, the ability to perceive the depth of an object relative to an observer, arises from the binocular sensory fusion of slightly dissimilar retinal images that have horizontal disparity by virtue of the lateral separation of the two eyes.^1^^,^^2^ Stereoacuity, reported in minutes or seconds of arc, describes the smallest horizontal disparity in binocular images that leads to the perception of depth in the observed stimulus. In observers with equal normal visual acuity and normal vision development, stereoacuity thresholds are typically of 20 to 40 seconds of arc.^2^^–^^4^ Stereoacuity will be worse when there are significant differences in the retinal images between eyes, that is, if the dichoptic images differ in contrast, size, or clarity.^5^^–^^9^ Differences in retinal image clarity and size can occur if there is unequal refractive error (anisometropia) or ocular media obstruction from corneal or lens anomalies, with both blur and contrast differences known to degrade stereoacuity.^10^ Stereoacuity also requires accurate ocular alignment so that the dichoptic images stimulate corresponding retinal areas for the perception of fused single vision.^2^

Nonconcordance of retinal images from ocular media obstruction, high anisometropic refractive error or strabismus early in life will interrupt the neurodevelopment of the visual system and cause amblyopia.^11^ Patients with amblyopia may have nonrecoverable reduced visual acuity, may suppress or ignore the visual signal from one eye and are likely to have reduced or absent stereopsis (binocular depth vision).^12^^–^^15^ In addition to visual acuity and stereoacuity loss, amblyopia results in impaired position discrimination and contrast sensitivity^11^ and, furthermore, may be associated with higher order functional and processing impairments that suggest visual neurodevelopmental retardation beyond the primary visual cortex.^16^^–^^18^

Interocular suppression of visual information from the amblyopic eye is frequently reported in amblyopia,^13^^,^^19^ presumably to compensate for conflicting visual input.^20^ As well as limiting stereopsis by preventing sensory fusion,^21^ the role that suppression plays in causing or limiting amblyopia recovery is yet to be fully determined. Novel treatments for amblyopia are aimed at reducing suppression of the amblyopic visual pathway to improve binocularity^22^^–^^25^; however, the relative contribution of interocular differences in visual acuity and depth of suppression in determining the limit of stereoacuity is unclear. For example, it has been believed for some time that amblyopia (reduced acuity in one eye) is the primary problem and loss of binocularity, the consequence. However, more recently it has been suggested that it is the other way around^13^^,^^26^^,^^27^; loss of binocularity is the primary problem and amblyopia the consequence. According to the former explanation one would expect an inverse relationship between suppression and acuity loss; the more amblyopic the eye, the less it needs to be suppressed to ensure it does not interfere in binocular vision. On the other hand, the latter explanation would assume a direct relationship, whereby the greater the suppression the greater the resultant amblyopia. A number of studies have shown that there is a direct relationship between suppression and acuity loss in amblyopia.^4^^,^^15^ Quite apart from these interrelationships in amblyopia, it would be of value to know the extent to which sensory eye dominance and stereopsis are correlated in the normal binocular population and the extent to which degraded monocular input affects ocular sensory dominance and stereopsis.

In this study we seek to make a distinction between simulations of acuity-like losses and suppressive-like losses. From what we know about the contrast sensitivity deficit in amblyopia,^28^^–^^30^ the higher the spatial frequency, the greater the loss. It is like a lowpass filtering, and the resultant loss in acuity would be simulated by an increased slope of the amplitude spectrum. We use the Bangerter filters in normal participants to simulate the amblyopic loss.^31^^,^^32^ Suppression affects low as well as high spatial frequencies^33^ and is best simulated by a vertical translation of the 1/f amplitude spectrum line. It is the standard metric used to quantify suppression in the case of amblyopia.

With the advent of polarized or three-dimensional monitors, new dichoptic tests have been developed that measure stereoacuity to threshold,^3^^,^^25^^,^^34^ and tests can also be used to quantify the relative contrast balance required for binocular perception (a measure of the depth of suppression).^19^^,^^35^^,^^36^ The contrast of stimulus can be modified in the dichoptic displays to attenuate presentations to either eye so the factors influencing stereoacuity threshold can be investigated. With this method contrast is equally reduced across all spatial frequency components.

In this study stereoacuity of adults with abnormal binocular vision development from strabismus and/or amblyopia was measured and interactions between the interocular sensory dominance (depth of suppression), visual acuity decrement (degree of amblyopia) and stereoacuity threshold determined. To mimic monocular visual acuity impairment Bangerter filters were introduced before one eye of observers with normal visual development; the impact of the reduced visual acuity and sensory dominance imbalance on stereoacuity was determined. Additionally, the monocular contrast attenuation feature of the digital stereoacuity test was used to separately measure the effect of interocular contrast difference on stereoacuity thresholds. The prediction was that if the main determinant of stereopsis was monocular acuity loss rather than interocular suppression, then stereoacuity data with the Bangerter filters (our simulation of the monocular acuity loss) should be more correlated with stereoacuity than with monocular contrast attenuation (our simulation of the interocular suppressive imbalance). In the case that stereopsis is more affected by the binocular suppressive imbalance, then the reverse would be the case; stereoacuity would be more impacted by the interocular contrast difference. Finally, stereoacuity was retested in a subset of amblyopic participants with the contrast of the stimulus presented to the dominant eye reduced, proportional to the determined interocular dominance, to counter suppression. For amblyopic participants the prediction was that if stereopsis were limited mainly by suppression, then by neutralizing suppression by altering the contrast, stereoacuity would be expected to improve.

This study had three aims: (1) to determine the relative strength of association between reduced visual acuity, the depth of suppression (contrast balance) and the stereoacuity in observers with abnormal binocular vision development (this directly bears on the etiology), (2) to determine the relative contribution of induced visual acuity or interocular dominance deficits to stereoacuity loss when vision is monocularly impaired in observers with normal vision development, and (3) to determine whether stereoacuity improves in amblyopic observers when interocular dominance balance is altered to neutralize suppression.

## Methods

### Participants

Adult participants were prospectively recruited for a study of binocular vision perception. All had a comprehensive vision and intraocular health exam (previous treatment history, visual acuity (electronic Early Treatment of Diabetic Study e-ETDRS and near logMAR), clinical stereoacuity (Randot Preschool Stereoacuity Test (RPST); Stereo Optical Co, Inc., Chicago, IL, USA), ocular alignment (prism alternating cover test) and Worth 4 Dot response at 33 cm to confirm eligibility criteria. Classification criteria for abnormal binocular vision group were a history of strabismus and/or amblyopia (anisometropic, strabismic or combined mechanism). Amblyopia was defined as an interocular difference in best-corrected visual acuity of 0.2 logMAR or worse. Inclusion criteria for the normal binocular vision group were best-corrected visual acuity of 0.0 logMAR or better, and 60 sec of arc stereoacuity on RPST. None of the participants had coexisting general developmental, systemic, or ocular pathology or congenital abnormality.

The study was conducted in accordance with the requirements of the Queensland University of Technology Human Research Ethics Committee. All participants were given a full explanation of the experimental procedures and written informed consent was obtained. All protocols were in accord with the guidelines of the Declaration of Helsinki.

### Apparatus

#### Digital Random-Dot Stereogram

A modified random dot computer displayed stereogram (McGill Vision Research Institute) was used to determine stereoacuity, reported as the minimum angle of binocular disparity at which the stereoscopic image was correctly identified (range of 1–4096 arc sec).^3^ Stimuli were viewed through synchronized shutter glasses (Nvidia 3D Vision 2–Model P1431) on 24″ Asus VG248QE 3D monitor (Nvidia Corporation, Santa Clara, CA, USA) so different targets could be presented to each eye. Before commencing the test, participants used an inbuilt alignment feature to horizontally and vertically align the two images. Stimuli were bandpass random dots (isotropic log-Gabor, size 10 arc minute, mean luminance 48 cd/m^2^) against a uniform gray background (34 cd/m^2^). A pie-shape was created by modulating the disparity of the dots that when observed with the shutter glasses gave the percept of a 10-degree floating circle with a missing sector. Participants reported the perceived position of the missing sector using a four alternative forced choice procedure.

The disparity of the stimulus presented in each trial was controlled by a pair of interleaved staircases.^37^ One staircase had a two-down/one-up rule, and the other a one-down/one-up rule. On each trial, one of the two staircases was randomly selected to control the disparity. Each staircase terminated after reaching 70 trials or nine reversals (whichever happened first). Thresholds were determined from the data by psychometric function fitting. The probability of responding correctly for each disparity was plotted on a base-2 logarithmic disparity axis. Psychometric functions were fit by a cumulative normal function using Palamedes.^38^ The log_2_ disparity value at 62.5% correct gave a log-transformed stereoacuity threshold.^3^ The stimulus generation and properties of this test have been recently fully described, with repeatability and validity determined in children and adults with normal and abnormal binocular vision.^27^

The Michelson contrast (range 1%–100%) of stimulus could be varied for the presentations to either eye. Baseline measurements were conducted at a contrast of 80%. The background luminance was set to 101 cd/m^2^. At baseline, the contrast of stimulus presented to the nondominant/dominant eye was set to equal (1:1). In Experiment 2, contrast of the stimulus presented to the dominant eye was reduced in 10 percent increments. In Experiment 3, contrast was adjusted based on the participant's contrast ratio (CR) such that the ratio of stimulus contrast presented to the nondominant/dominant eye was proportional to the depth of suppression.

#### BF Score

BF score was used to assign an ordinal rank^35^ to grade binocular function in those participants with nil stereo recorded by RPST. The best RPST stereoacuity level correctly identified by the participant was converted to a log value providing a rank score from 1.6 (log 40 arc sec) to 3.3 (log 2000 arc sec). A score of 4 was assigned when there was no stereoacuity and the Worth 4 Dot test outcome indicated simultaneous perception or second-degree fusion. A score of five was assigned when there was no stereoacuity and the Worth 4 Dot test outcome indicated suppression.

#### Contrast Ratio (CR)

Interocular sensory dominance threshold was determined by measuring the contrast ratio (CR) between dichoptically presented letters at which it is equally likely that the observer will report the left or right eye letter.^19^^,^^39^ CR of 1.0 would indicate right eye (RE) and left eye (LE) had equal contrast letters when it was equally likely that a RE or LE letter was reported. Higher CR indicates that the nondominant eye required higher contrast of letters than the dominant eye to result in equal likelihood of reporting the dichoptic letters.

#### Bangerter Filters

Bangerter filters (Fresnel Prism and Lens Co., Eden Prairie, MN, USA) are a series of adhesive diffuse filters that introduce mild to moderate reduction in visual acuity.^40^ Bangerter filters (Ryser Optik, St. Gallen, Switzerland) are available in 0.1 to 0.2 ordinal steps from 1.0 (∼20/20) to 0.1 (∼20/200) and then LP, where the number is intended to represent the ordinal level to which visual acuity is reduced. Their optical properties have been characterized; they consist of microbubbles the density of which related to the image degradation (Pérez et al., 2010). The 0.3, 0.4 and 0.6 filters are not that different in terms of the effect on spatial frequency (Pérez et al., 2010).

It is stated that Bangerter filters are similar to a Gaussian filter in that they produce essentially monotonically decreasing contrast with increasing spatial frequency.^32^ For example, a 12 cyc/deg target log contrast sensitivity is reduced by 0.3 for Bangerter filter 0.8, increasing to 1.0 reduction for Bangerter filter 0.3 (Pérez et al., 2010). Contrast sensitivity assessed using Pelli-Robson charts is reduced for all Bangerter filters, but the effect is small until the 0.1 filter.^40^ The results of Li et al.^31^ show a selective effect for high spatial frequencies as BF density increases.

Hence, our decision to not use all available Bangerter filters. The thin plastic filters were cut to the trial lens size and placed on the front surface of a plano lens; 0.8, 0.6, 0.4, 0.1 and <0.1 (<0.1 was created by applying both the 0.4 and 0.1 filter).

### Study Design

#### Experiment 1: Amblyopic Observers

Visual acuity (VA), contrast ratio (CR), BF score based on RPST or Worth 4 Dot response and digital stereoacuity were determined in adult participants with abnormal binocular vision development from amblyopia and/or strabismus. Correlation and multiple regression analyses were used to assess interactions between interocular VA difference, CR, and BF score and stereoacuity.

#### Experiment 2: Monocularly Impaired Normal Observers

Adult observers with normal binocular vision development had VA, CR and stereoacuity measured at baseline and through a series of monocular Bangerter filters that degrade visual input. Stereoacuity was also measured through a range of monocular contrast attenuation of the stimulus viewed by the dominant eye. An initial practice run was conducted to familiarize the observer with the CR and stereoacuity tasks, then the order of test conditions was randomized between participants to limit the influence of a learning effect. The relative contribution of CR and interocular VA difference to stereoacuity was calculated.

#### Experiment 3: Contrast Rebalanced Stereoacuity in Amblyopic Observers

Stereoacuity was determined in a subset of amblyopic participants with (1) equal contrast of stimulus presented to dominant and nondominant eye, (2) the contrast of the stimulus presented to the dominant eye reduced proportional to the determined contrast ratio to neutralize suppression of the nondominant eye stimulus, (3) dominant eye contrast reduced by CR+25%CR, and (4) dominant eye contrast reduced by CR−25%CR. The order of presentation of the four contrast conditions was randomized between participants. Because this was our first exploration of how manipulating contrast would affect stereoacuity we decided to test either side of the CR by an amount that would be based on each participants CR rather than a fixed contrast.

### Statistical Analysis

Correlation coefficients were calculated to explore the relationships between VA and CR and stereoacuity. Nonparametric tests were used for ordinal data (BF Score). General linear multiple regression models were used to determine the strengths of relationships between CR and VA and stereoacuity threshold. Repeated measures analysis of variance (ANOVA) was used to determine effect of monocular contrast reduction on stereoacuity and quantify the change in stereoacuity, VA and CR with introduced monocular degradation (Bangerter filter). Post-hoc tests for pairwise comparisons used the Bonferroni correction for multiple comparisons. Paired *t*-tests were used to compare stereoacuity threshold at baseline and post rebalance of interocular contrast in the subset of participants with abnormal binocular vision. SPSS was used for statistical analyses (IBM SPSS Statistics for Windows, Version 25.0. Armonk, NY, USA)

## Results

### Experiment 1: Observers With Abnormal Vision Development

Twenty-one observers with a history of abnormal binocular vision development from strabismus, anisometropia, or both participated in experiment 1 (mean age 27 ± 11, range 21–63 years). Clinical details and previous strabismus and/or amblyopia treatment including refractive error correction are provided in the Supplementary Table. Refractive error correction was worn during all tests. One participant with high anisomyopia did not habitually wear refractive correction; all others had worn the refractive correction for more than six months before the study. Interocular VA difference between eyes ranged from 0.02 to 0.96 logMAR (mean 0.36 ± 0.30), with 13 participants amblyopic (interocular VA difference 0.20 logMAR or more). CR ranged from 1.29 to 39.16 (mean 9.62 ± 11.27). Binocular function ranged from 40 seconds on RPST to suppression on Worth 4 Dot test. BF score ranged from 1.6 to 5 (mean 3.14 ± 1.12). Fourteen of the 21 abnormal vision development participants had stereoacuity measurable with the digital random-dot test, with thresholds ranging from 40 to 3141 sec arc (mean 680 ± 948 sec arc); of these nine were amblyopic.

**Figure 1.**
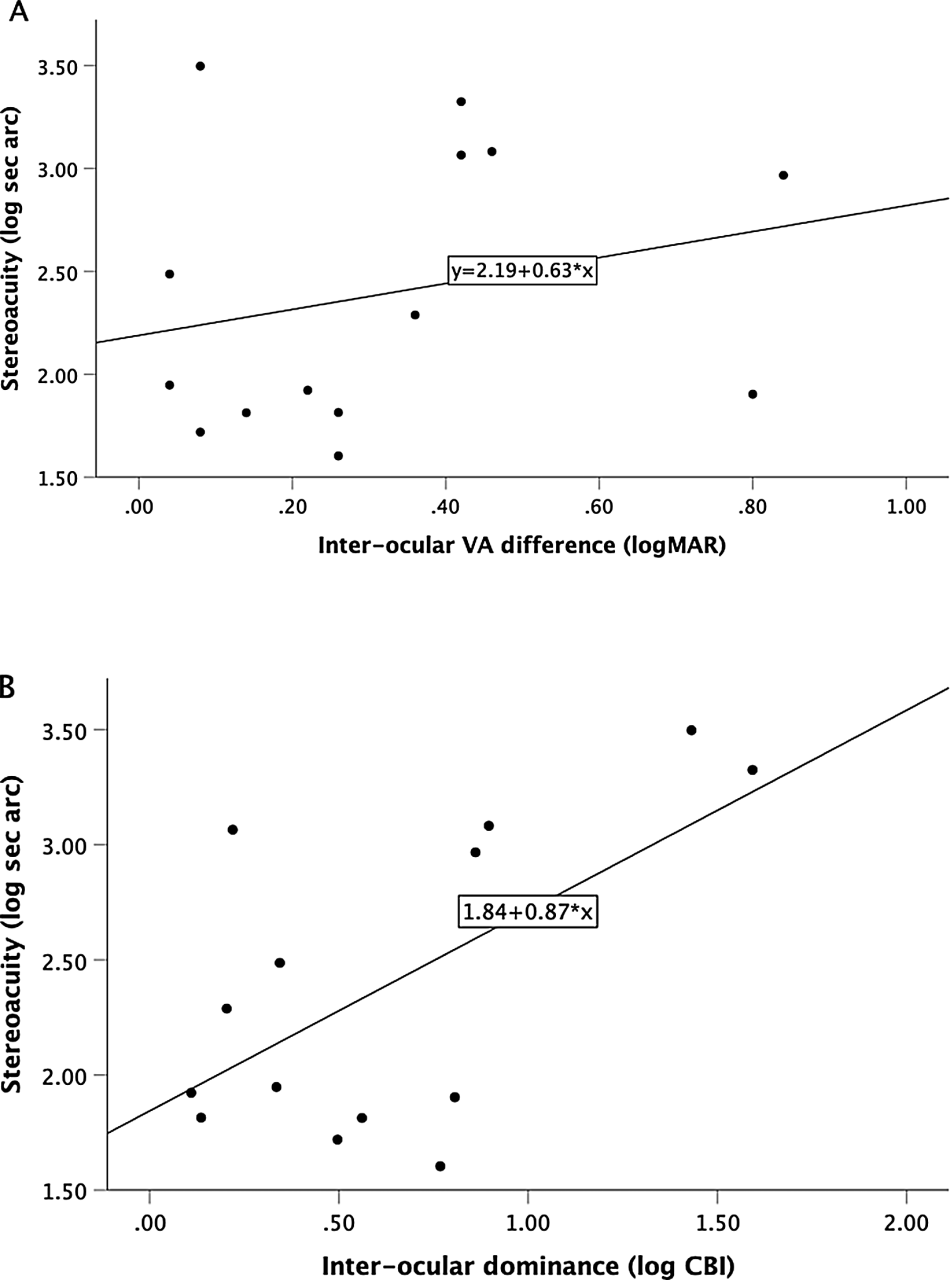
Variation in stereoacuity with (**A**) interocular difference in VA (*r* = 0.051, *P* = 0.863) and (**B**) interocular dominance ratio (CR AE/FE) (*r* = 0.821; *P* < 0.001) in observers with abnormal vision development from strabismus or amblyopia (n = 14).

BF score highly correlated with CR (*r*_s_ = 0.740; *P* < 0.001), but did not correlate with interocular VA difference (*r*_s_ = 0.184; *P* = 0.425) (n = 21). Digital stereoacuity (n = 14) also highly correlated with CR (*r* = 0.821; *P* < 0.001), but not with interocular VA difference (*r* = 0.051, *P* = 0.863) (Figs. 1A, 1B). Similar correlations were found in the amblyopic subgroup: BF score correlated with CR (*r*_s_ = 0.868; *P* < 0.001), but not interocular VA difference (*r*_s_ = 0.493; *P* = 0.087) (n = 13); digital stereoacuity (n = 9) correlated with CR (*r* = 0.775; *P* = 0.014), but not with interocular VA difference (*r* = 0.189, *P* = 0.626).

Multiple regression modeling showed that poorer stereoacuity was associated with contrast ratio (*P* = 0.001), but not with visual acuity decrement (*P* = 0.943). The multiple regression results indicated that 61.5% of the variance in stereoacuity could be explained by the model, which was significant (F_(2,11)_ = 11.40, *P* = 0.002).

### Experiment 2: Monocularly Impaired Normal Observers

Nineteen observers with normal binocular vision development participated in experiment 2 (mean age 31 ± 13, range 21–64 years). Interocular VA difference between eyes ranged from 0.00 to 0.10 logMAR (mean 0.02 ± 0.04) and CR ranged from 1.03 to 2.19 (mean 1.45 ± 0.33). All perceived 40 sec arc on RPST and digital stereoacuity ranged from 15 to 73 arc sec (mean 40 ±14).

### Attenuation of Monocular Contrast Input

**Figure 2.**
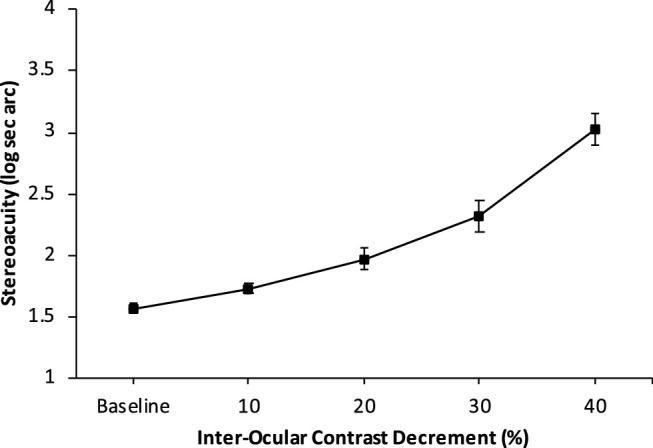
Change in stereoacuity threshold with progressive attenuation of dominant eye stimulus contrast. Baseline indicates equal contrast of stimulus presented to RE and LE. 10 indicates 10% reduction in contrast of stimulus presented to dominant eye, 40 indicates 40% reduction in contrast of stimulus of presented to dominant eye (mean; error bars represent ±1 SE).

The monocular contrast attenuation feature of the digital stereoacuity test was used to separately measure the effect of interocular contrast differences on stereoacuity thresholds. The contrast of the stimulus presented to the nondominant eye was held constant whereas the contrast of the stimulus viewed by the dominant eye was reduced in 10% increments. Stereoacuity was not able to be reliably determined if dominant eye contrast was reduced greater than 40%. Stereoacuity progressively declined from baseline with introduced contrast reduction of the stimulus viewed by the dominant eye (RM ANOVA; F_(9.09, 50.61)_ = 71.65; *P* < 0.001) (Fig. 2). Log Stereoacuity highly associated with reduction in dominant eye stimulus contrast (R^2^ = 0.920) with linear regression showing 0.35 log stereoacuity increase in threshold per 10% contrast decrement. Pairwise comparison with Bonferroni correction for multiple comparisons showed that each change in contrast produced a significant stepwise increase in stereoacuity threshold. (Table 1)

**Table 1.** Stereoacuity at Baseline and With Progressive Decrement of Dominant Eye Stimulus in the Dichoptic Random-Dot Stereoacuity Display

% Difference in Dichoptic Contrast	Stereoacuity, Sec Arc Mean (SD)	Relative Change From Baseline
0	40 (16)	1
10	57 (22)^*^	1.4^*^
20	132 (212^*^)	5.3^*^
30	512 (750)^*^	12.8^*^
40	1780 (1360)^*^	44.5^*^

Mean (Std Dev) and the relative change from baseline (condition outcome/baseline outcome).

* Indicates significant change from baseline (*P* < 0.01 pairwise comparison with Bonferroni correction for multiple comparisons).

### Effect of Bangerter Filters on Visual Acuity and Contrast Ratio and Stereoacuity

Introduction of a Bangerter filter before the dominant eye caused a difference in VA between eyes (F_(3.28, 58.5)_ = 266; *P* < 0.001), with all filter conditions significantly reducing VA from that measured as baseline. CR increased with the filter conditions (F_(1.43, 20.57)_ = 17.76; *P* < 0.001), with CR significantly poorer than baseline for filter conditions 0.4, 0.1 and < 0.1. Stereoacuity threshold increased with monocular BF filter (F_(3.66.26)_ = 16.85, *P* < 0.001); however, from pairwise comparison with Bonferroni correction for multiple comparisons, significant change from baseline only with BF filter < 0.1 (both 0.1 and 0.4 were applied) (Table 2).

**Table 2.** VA, CR and Stereoacuity at Baseline and With Monocular Degradation by Bangerter Filter

	Interocular VA		CR		Stereoacuity	
Bangerter Filter	Mean (SD)	Relative Change From Baseline	Mean (SD)	Relative Change From Baseline	Mean (SD)	Relative Change From Baseline
Baseline	0.02 (0.04)	1	1.44 (0.32)	1	40 (16)	1
0.8	0.12 (0.10)^*^	6	1.49 (0.41)	1	40 (17)	1
0.6	0.29 (0.10)^*^	14.5	1.87 (0.93)	1.3	41 (17)	1
0.4	0.48 (0.16)^*^	24	2.44 (1.08)^*^	1.7	48 (14)	1.2
0.1	0.54 (0.09)^*^	27	3.64 (1.59)^*^	2.5	46 (14)	1.2
<0.1	0.97 (0.12)^*^	48.5	9.84 (8.71)^*^	6.8	88 (36)	2.2^*^

Mean (std dev) and the relative change from baseline (condition outcome/baseline outcome).

* Indicates significant change from baseline (*P* < 0.01 pairwise comparison with Bonferroni correction for multiple comparisons).

The relative change in VA, CR, and stereoacuity were normalized to the baseline measure to display the relative rate of visual function loss with the filter conditions. VA shows the greatest degradation with filters, while both CR and stereoacuity remained relatively robust until the denser filter combination was introduced.

**Figure 3.**
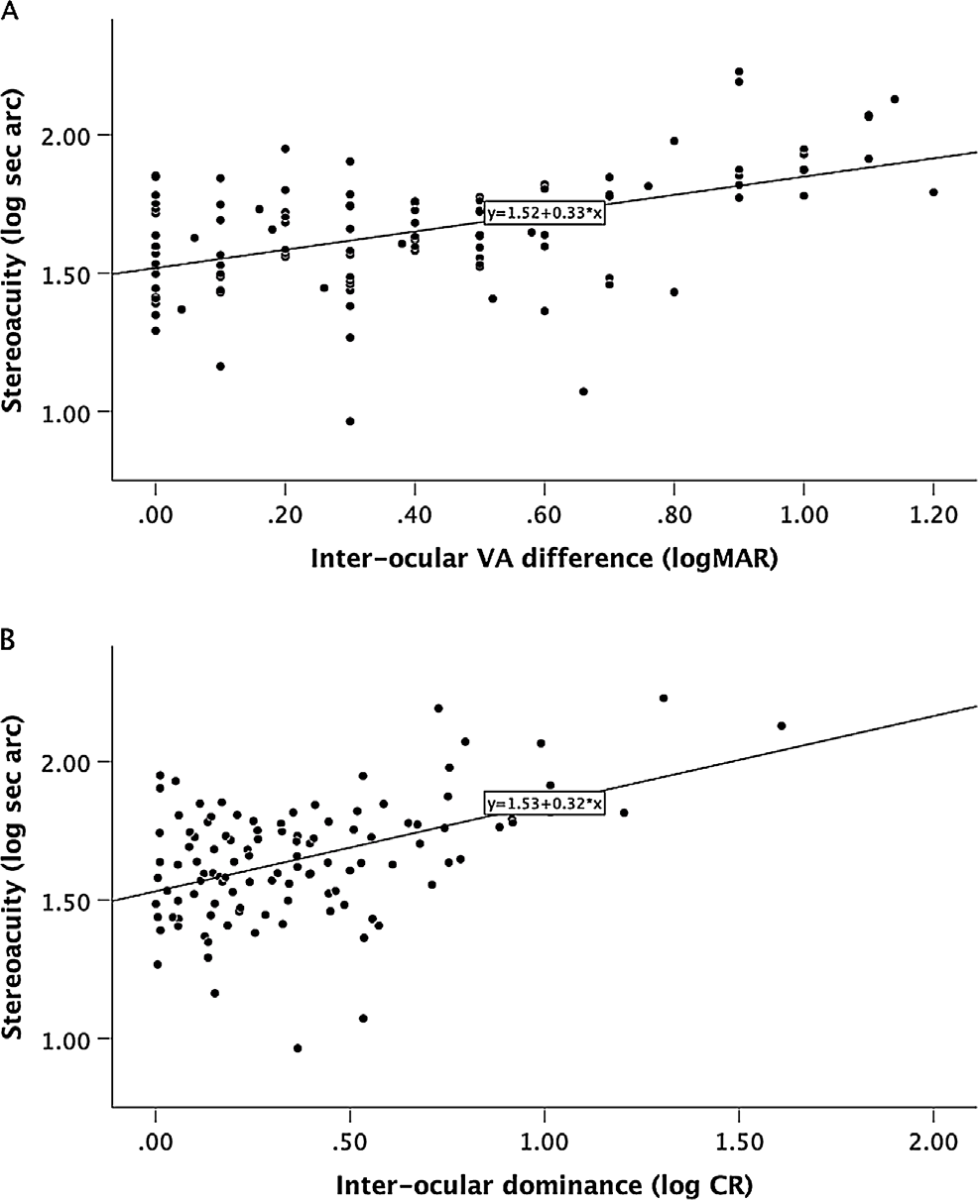
Variation in stereoacuity with Bangerter filter induced (**A**) interocular visual acuity difference (*r* = 0.515, *P* < 0.001) and (**B**) interocular dominance ratio (CR impaired/nonimpaired eye) (*r* = 0.465, *P* < 0.001) in observers with normal binocular vision development.

The Bangerter filter conditions provided a data set of 114 measures of stereoacuity, VA decrement and CR. Stereoacuity significantly correlated with both induced interocular VA difference (*r* = 0.515, *P* < 0.001) and CR (*r* =0.465, *P* < 0.001) (Figs. 3A, 3B). Multiple regression analysis was used to test the relative contribution of VA decrement and interocular dominance decrement to stereoacuity threshold. The multiple regression results indicated that 30% of the variance in stereoacuity could be explained by the model, which was significant (F_(2,111)_ = 24.45, *P* < 0.001). Poorer stereoacuity threshold associated with both contrast ratio and visual acuity decrement (*P* < 0.001). Inclusion of the interaction between VA and CR did not change the R squared of the model, indicating that the joint effect was not greater than the sum of the main effects.

### Experiment 3: Dominance Rebalanced Stereoacuity in Amblyopic Observers

**Figure 4.**
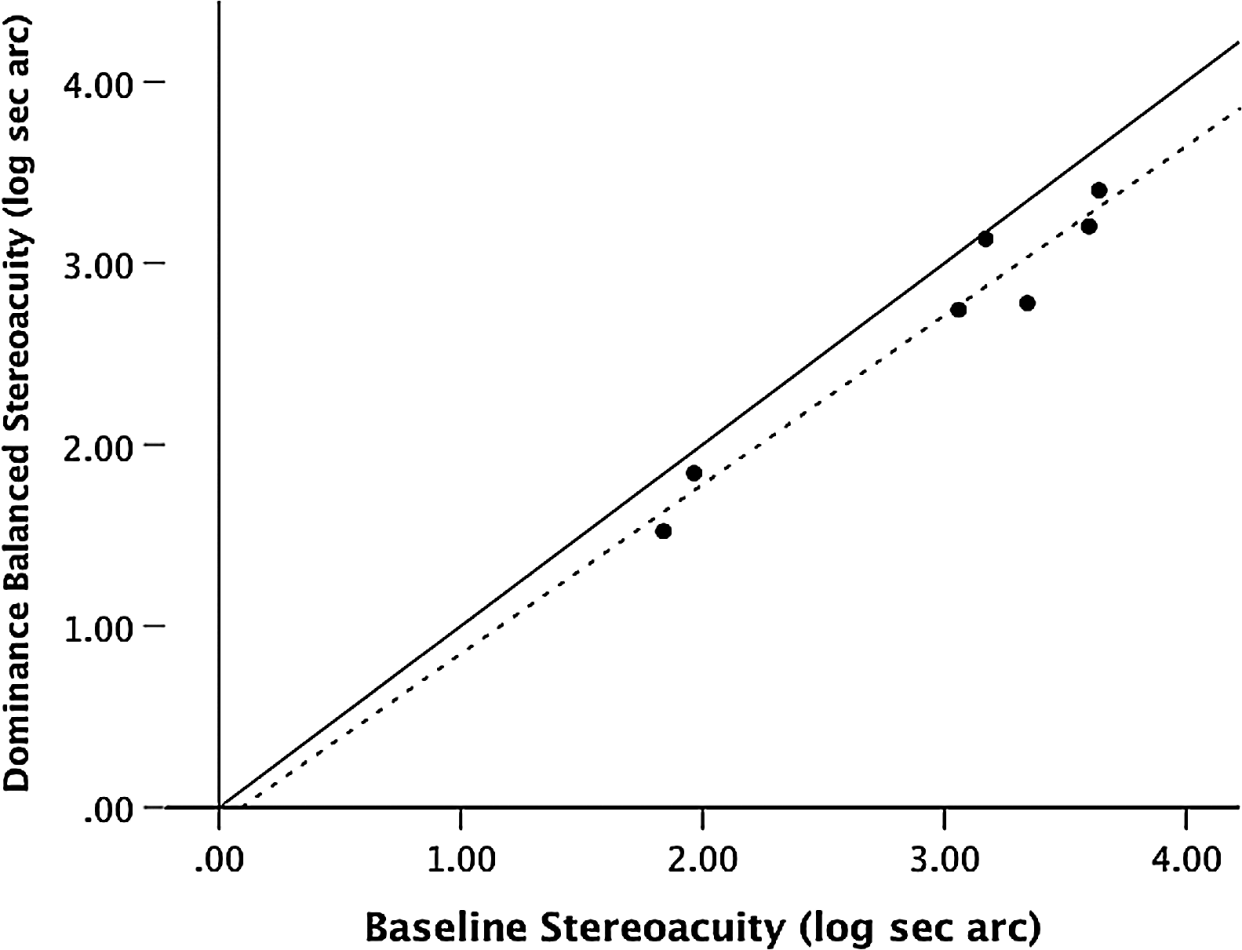
Threshold stereoacuity before versus after interocular balance adjustment based on individual CR. The *dashed line* is the best-fit line for the data. Points that lie on the *solid unity line* indicate no change in threshold. All points that lie below the unity line represent improved stereoacuity when dominance balance is altered.

Seven of the abnormal binocular vision observers from Experiment 1 participated in Experiment 3, which was conducted at a subsequent session. Stereoacuity significantly improved when the contrast was reduced to the dominant eye in proportion to the measured CR (paired t-test p=0.005). Table 3 provides the individual amblyopic observer's data including stereoacuity threshold for contrast attenuation by CR ± 25% CR. The shaded data from Table 3 of stereoacuity pre and post dominance balance adjustment are plotted in Figure 4. Points that lie on the unity line indicate no change in threshold. All points that lie below the unity line represent improved stereoacuity when contrast balance is altered to neutralize suppression.

**Table 3.** Individual Amblyopic Observer Stereoacuity at Baseline and With Dominant Eye Contrast Reduced Proportional to Dominance Ratio (CR)

			Dominance Balanced Stereoacuity (Sec Arc)		
Observer	CR	Baseline Stereoacuity (Sec Arc)	CR	+25%CR	−25% CR
1	4.85	92.22	69.64	73.88	60.74
2	5.553	68.89	33.31	32.12	45.15
3	91.5926	4359.21	2522.58	2788	1782.59
4	2.5828	1145.63	553.14	903.56	647.12
5	4.0378	3964.91	1595.73	2087	2053.26
6	39.1606	2204.13	602.31	1054.45	881.7
7	42.4783	1482.48	1360.97	1556.76	1478.09

## Discussion

Loss of stereopsis is recognized as the principal visual impairment that accompanies amblyopia under ordinary binocular viewing conditions, with flow on effects to visuomotor proficiency and potentially limiting vocational options.^12^^,^^31^^,^^32^ Emerging binocular treatments for amblyopia target interocular sensory dominance imbalance to recover sensory fusion and stereopsis^20^^–^^22^^,^^33^; however, the basis of this loss is not well understood. To better understand the reason for this loss, one needs to know the relationships between severity of monocular vision loss, interocular sensory dominance and stereoacuity. In this study, stereoacuity of adults with amblyopia was measured, and interactions between depth of suppression, visual acuity decrement (degree of amblyopia) and stereoacuity threshold were determined. Poor stereoacuity highly correlated with depth of suppression, but did not relate to the VA decrement that is typically used to define the severity of amblyopia.

The lack of clear relationship between stereoacuity and VA, particularly in strabismic amblyopia, has been well established.^12^ Reductions in VA and interocular dominance are highly related, so it is difficult to disentangle the relative contributions of each to stereoacuity.^15^ These known interrelationships were controlled for by examining stereoacuity outcome in a multiple regression model that took into account the intercorrelation that exists between measures of vision loss. In the amblyopic group, depth of suppression clearly emerged as the most important factor impacting on stereoacuity, even though the strength of this relationship relied on the results of our two most severe cases, because we have no independent reason for doubting their validity. The strong relationship between the contrast balance ratio and the clinically derived BF score in binocularly abnormal observers with deepest suppression, previously reported^35^ and seen in the current data, supports the inclusion of these more severe examples.

It is well known that blurring, filtering, or reducing luminance or contrast to one eye reduces stereoacuity in people with normal vision^8^^,^^12^; however it appears that the main determinant of stereo loss in amblyopia is the depth of suppression. To obtain more insight into how the monocular deficits in amblyopia affect stereo performance, the relative effect of interocular dominance and interocular acuity loss on stereoacuity was examined in nonamblyopic observers with the use of Bangerter filters. The monocular contrast attenuation feature of the threshold stereoacuity test was used to model the effect of interocular dominance imbalance on stereoacuity. Clarity of images presented to each eye did not change while the contrast of stimulus seen by the dominant eye was progressively attenuated, modeling increased severity of interocular suppression. Stereoacuity was highly sensitive to the introduced imbalance of dominance; with 0.35 log sec arc increase in threshold per 10 percent reduction of dominant eye stimulus contrast.

Bangerter filters are known to degrade VA, vernier acuity and will also reduce contrast sensitivity at higher spatial frequency.^30^^,^^35^ Although Bangerter filters degrade vision mainly by a loss of contrast at high spatial frequencies possibly as a consequence of light scatter, it has been suggested that there is also a phase scrambling of image features as a consequence of their microelement composition.^31^ In agreement with previous reports, the filters had progressive, profound consequence on optotype acuity, probably by a combination of amplitude and phase filtering. However, the filters did not significantly impact stereoacuity until the very highly degrading filters that reduce visual acuity to 1.0 logMAR on average were in place. The phase scrambling effect would be expected to impact stereo sensitivity by decorrelating left and right eye images.^41^ Little impact was seen in this study because our random element size in our stereo test was relatively large, some 100 times larger than for a previous study^31^ where the impact of Bangerter filters on stereo sensitivity was found to be profound. Our simulation of amblyopia using Bangerter filters where there is an element of monocular phase scrambling is plausible because there is a large literature detailing the spatial scrambling that occurs in the amblyopic eye.^42^^–^^50^ The conclusions that can be drawn from our simulation include 1. Stereoacuity and visual acuity are not always tightly linked and 2. Although amblyopes exhibit spatial scrambling, the decorrelation that this produces is not enough to explain their poor stereo performance. Thus it is likely that neither their reduced acuity nor their spatial uncertainty provide a satisfactory explanation for their poor stereopsis. However, stereoacuity was sensitive to the reduction of stimulus contrast to the dominant eye in normal (simulated suppression) and correlated with the depth of suppression in amblyopes, suggesting a more plausible explanation for their three-dimensional deficiency.

In our final experiment, stereoacuity was retested in a subset of amblyopic participants with the contrast of the stimulus presented to the dominant eye reduced, proportional to the determined contrast ratio, to neutralize suppression. Small, but significant, improvements were seen, with no participant having a stereoacuity finding that was poorer than baseline with contrast rebalanced conditions. For most observers, stereoacuity was best with contrast reduced in the dominant eye proportional to their determined CR, rather than when contrast was varied by plus or minus 25% of CR. Our finding supports the general conclusions of psychophysical and physiological studies that binocular interactions are present but dormant in amblyopia.^51^

Collectively, our findings suggest that the extent of suppression is the main visual limitation that influences reduced stereopsis in participants with a history of abnormal vision development from strabismus or amblyopia. Prior studies have shown anomalous interactions between the eyes of amblyopic observers that can be changed by manipulation of the relative information presented to each eye.^37^ Here we show that this will impart an instantaneous improvement in stereoscopic depth perception, conveying a functional advantage if amblyopia treatment leads to reductions in suppression.

In summary, our experiments show that stereoacuity is relatively robust when observers with normal vision development have substantial monocular blur and spatial scrambling, compared to contrast-based suppression. In amblyopic observers, the extent of suppression is the main visual limitation that influences reduced stereopsis. Observers with amblyopia have more severely degraded stereoacuity than would be predicted from models where a monocular input is amplitude or phase filtered, supporting the evidence for an additional neural limitation that we argue here is interocular suppression. Inclusion of the interaction between VA and CR did not change the R squared of the model, indicating that the joint effect was not greater than the sum of the main effects.

This study provides further evidence for intact binocular mechanisms in amblyopia that may be promoted by conditions that target simultaneous binocular perception, with demonstration that stereopsis improves when suppression is neutralized. Prior studies have shown anomalous interactions between the eyes of amblyopic observers can be changed by manipulation of the relative information presented to each eye.^37^ Here we have shown that this can recover stereoscopic depth perception to some degree, conveying a functional advantage if amblyopia treatment leads to reduced suppression. These findings provide support for binocular treatment regimens designed to alleviate suppression to address the visual losses of amblyopia, particularly the binocular ones.

## Supplementary Material

Supplement 1
