# Supplementary material for: Suppression Rather Than Visual Acuity Loss Limits Stereoacuity in Amblyopia
Source: Invest Ophthalmol Vis Sci. 2020 Jun 24;61(6):50. doi: 10.1167/iovs.61.6.50 (PMC7419721; doi:10.1167/iovs.61.6.50)
Supplement: Supplement 1 [file iovs-61-6-50_s001.pdf]

Observer	Age	Binocular Vision Condition	Habitual Refraction	VA OD (logMAR)	VA OS (logMAR)	History
01	25	R ET	R - L -	0.42	0.0	Patching + VT
02	24	R ET + A	R -5.00/-3.00x180<br>L plano/-2.00x180	0.72	0.1	EOM surgery
*03	24	A + M	R +3.00 L plano	0.02	-0.12	NIL
04	27	A	R - L -	0.0	0.84	Trauma to L eye as a child
05	23	M	R - L -	-0.08	0.38	NIL
06	21	A	R - L -	0.12	-0.1	NIL
*07	21	A	R - L -	-0.02	0.78	NIL
08	26	R XT	R - L -	1.04	0.08	NIL
*09	24	L HTET	R +0.75/-0.25x10<br>L +1.25/-0.25x160	-0.02	0.06	EOM surgery OU at 2yrs of age, OS at 17yrs + patching
*10	21	A + L XT	R +0.25/-0.75x170<br>L -9.50/-4.50x20	-0.04	0.44	NIL
11	26	ALT ET	R - L -	-0.02	0.14	EOM surgery at 3yrs of age
12	63	R ET	R - L -	0.8	-0.02	Patching
13	21	BH	R +3.75<br>L +4.25/-0.75x25	0.0	0.36	Uncorrected hyperopia until 17 years of age, worse LE
14	27	DI	R - L -	-0.04	0.0	VT (Brock string)
15	27	A + L HTXT	R +0.25/-1.00x110<br>L +4.50/-2.50x74	-0.08	0.18	Corrected from 11-12yrs of age, then did not wear Rx again until 18yrs. Currently wears *Shaw* aniseikonic lenses.
16	27	L HTXT	R -1.25/-0.50x130<br>L -1.00/-0.50x55	-0.14	-0.12	NIL
17	54	INT L XT	R - L -	0.3	0.38	NIL
*18	24	ALT HTXT + A	R +2.00/-2.00x178<br>L +0.75/-1.50x175	0.08	0.0	Bilateral infantile ET, EOM surgery OU + patching
19	21	L HT	R - L -	-0.06	-0.02	Potential L CNIV palsy
*20	22	L HT XT + A	R +0.25/-2.00x170<br>L +3.00/-2.75x30	0.0	0.42	Bilateral infantile ET, EOM surgery OS at 6mths of age, then OD at 7yrs + patching
21	27	A + M	R +2.25/-1.00x2<br>L +1.00/-0.50x180	0.14	-0.12	NIL
*	27	S	R -2.35/-1.00 x 165<br>L -1.00/-1.25 x 5	0.02	-0.02	Strabismus surgery at 5 years of age

SUPPLEMENTARY. Clinical details for participants with abnormal binocular vision.
R, right; L, left; ET, esotropia; XT, exotropia; HT, hypertropia; ALT, alternating; INT, intermittent; M, microstrabismus; A, anisometropia; BH, bilateral hyperopia; DI, divergence insufficiency, VT, vision therapy.
